# Reconstruction of lossless molecular representations from fingerprints

**DOI:** 10.1186/s13321-023-00693-0

**Published:** 2023-02-23

**Authors:** Umit V. Ucak, Islambek Ashyrmamatov, Juyong Lee

**Affiliations:** 1 Research Institute of Pharmaceutical Science, College of Pharmacy, Seoul National University, 1 Gwanak-ro, Gwanak-gu, Seoul, 08826 Republic of Korea; 2 Department of Chemistry, Kangwon National University, Chuncheon, 24341 Republic of Korea; 3 Molecular Medicine and Biopharmaceutical Sciences, Graduate School of Convergence Science and Technology, Seoul National University, 1 Gwanak-ro, Gwanak-gu, Seoul, 08826 Republic of Korea

**Keywords:** Fingerprints, SMILES, SELFIES, Neural Machine Translation

## Abstract

The simplified molecular-input line-entry system (SMILES) is the most prevalent molecular representation used in AI-based chemical applications. However, there are innate limitations associated with the internal structure of SMILES representations. In this context, this study exploits the resolution and robustness of unique molecular representations, i.e., SMILES and SELFIES (SELF-referencIng Embedded strings), reconstructed from a set of structural fingerprints, which are proposed and used herein as vital representational tools for chemical and natural language processing (NLP) applications. This is achieved by restoring the connectivity information lost during fingerprint transformation with high accuracy. Notably, the results reveal that seemingly irreversible molecule-to-fingerprint conversion is feasible. More specifically, four structural fingerprints, extended connectivity, topological torsion, atom pairs, and atomic environments can be used as inputs and outputs of chemical NLP applications. Therefore, this comprehensive study addresses the major limitation of structural fingerprints that precludes their use in NLP models. Our findings will facilitate the development of text- or fingerprint-based chemoinformatic models for generative and translational tasks.

**Supplementary Information:**

The online version contains supplementary material available at 10.1186/s13321-023-00693-0.

## Introduction

The Simplified Molecular-Input Line-Entry System (SMILES) [1] is the most widely used linear representation for describing chemical structures. In SMILES, several simple rules are used to convert a chemical structure into a character string. This allows multiple unique SMILES strings to be used to represent molecules. Since its inception, SMILES has undergone various extensions [2–5], and among them, canonicalization algorithms, the integration of isotopism and the addition of stereochemical information (isomeric SMILES) are major milestones [6–9].

Although the simplified line notation of SMILES is superior to other one-dimensional representation schemes such as the Wiswesser Line Notation (WLN) [10], SYBYL line notation (SLN) [11], and International Chemical Identifier (InChI) [12], its internal structure leads to several problems when used in natural language processing (NLP) algorithms [13–15]. SMILES-based neural machine translation (NMT) models are prone to generate invalid SMILES strings [16, 17], which can be attributed to the fragile grammar (i.e., a strong dependence between tokens). Most notably, SMILES related issues seen in NMT models also occur in the most commonly used deep generative models such as variational autoencoders and generative adversarial networks that generate SMILES strings [18–21]. Because these models formulate predictions for one character at a time, a single-character alteration often suffices to invalidate an entire SMILES string. In addition, novel valid SMILES strings generated by AI-models are not guaranteed to be chemically valid.

To address the aforementioned problem, several attempts have been made to ensure the syntactic and chemical validity of the SMILES predictions  [22–27]. The challenges posed by the SMILES syntax have prompted the development of alternative syntaxes such as DeepSMILES [28] and SELF-referencIng Embedded strings (SELFIES) [29]. SELFIES is a new way of representing molecules that is receiving increasing attention from the scientific community and is being actively developed. Unlike SMILES, SELFIES units are enclosed by square brackets where no cuts is allowed within during tokenization, ensuring the generation of syntactically and semantically valid graphs. Multiple benchmarks have demonstrated that SELFIES outperforms alternative approaches in terms of validity and diversity of generated molecules.

The most commonly used NLP methods in chemistry are text generation and NMT. Particularly, these NLP methods aim to generate meaningful sequences from meaningful tokens. Therefore, tokenization is a pivotal preprocessing step in many NLP tasks. SMILES strings are meaningful as a whole, and any tokenization procedure must dissect these strings arbitrarily. From a chemist’s perspective, the atom-wise or character-wise tokenization of SMILES strings does not produce fully interpretable tokens. This is because many characters in SMILES strings correspond to topological characteristics, such as the digits in ring opening and closures, or parenthesis enclosing branches, that do not correspond to physical entities. In addition, most SMILES tokens are indistinguishable owing to their repetitiveness and simplicity. Considering that the primary design purpose of SMILES is to serve as a universal exchange format, it is understandable that interpretable insights cannot be derived from tokenization.

Despite the challenges mentioned above, SMILES representation plays a prominent role in chemical language modeling because they are preferred over the generation of a set of fingerprint features (incomplete description of a molecule), as the latter would require extensive database searches to identify matches and is therefore not desired. There are currently few studies in the literature using fingerprints as model outputs. In the field of molecular generation, Kadurin et al. [30] first proposed the use of an adversarial autoencoder to generate novel compounds for cancer treatment. They used MACCS keys (166-bit long binary vectors) as the input–output data structure, together with the inhibition concentration of the molecules. The model was trained on cancer cell line assay data, and the generated fingerprints were used to screen compounds on PubChem to identify candidate molecules with anticancer properties. In the field of reaction route planning, our previous works have shown that fragmental and topological descriptors can be effectively used as the input–output data structure in end-to-end NMT pipelines [31, 32].

Furthermore, interpretability necessitates the existence of meaningful tokens because NLP models tend to learn the relationships between these tokens. Thus, the interpretability of an individual token is highly desirable. However, the chemical interpretability of conventional NLP methods is hampered as SMILES representations are not fully interpretable from a chemical perspective. Indeed, SMILES is a highly efficient system for capturing information about molecular structures, and issues arise only when SMILES are tokenized. This contradicts the recent statement by Tu et al. [33], who propounded that SMILES is inefficient in capturing structural information because SMILES augmentation can provide additional performance gains [34]. As an alternative to SMILES representations, molecular fingerprints and substructural keys can be employed. They are designed to capture chemical features, concepts, or structural patterns, yielding an interpretable set of tokens suitable for NLP applications.

Several studies have recently explored the conversion of the extended-connectivity fingerprint ECFP [35] to SMILES representation. Within the context of data sharing and confidentiality, Le et al. [36] suggested the NeuralDecipher model. The model deduces the molecular structure of compounds using a two-step process involving a feedforward neural network model that predicts a compact vector representation of the compounds given their ECFP, and a pre-trained model that converts this representation into SMILES. NeuralDecipher showed a success rate of 69%. Kwon et al. [37] proposed a data-driven evolutionary molecular design methodology using a genetic algorithm, a recurrent neural network (RNN), and a deep neural network to evolve ECFP vectors of seed molecules and reconstruct chemically valid molecular structures in SMILES format. The model showed a success rate of 62.4%. Cofala and Kramer also used a genetic algorithm to demonstrate the ability to reconstruct molecules similar to the specified target or even the original molecule from ECFP representations [38]. Their method also showed a reconstruction rate of 58% $\sim$ 68%. Overall, these studies show the potential for using ECFPs as a starting point for generating molecular structures in SMILES representation, either through direct prediction or through genetic algorithms and evolutionary design techniques.

Because the construction of molecular fingerprints is a lossy procedure, the use of fingerprints leads to the generation of stand-alone interpretable tokens. Moreover, fingerprints are well suited to the attention mechanism because attention is a permutation-invariant operation [39]. Furthermore, attention-based models, such as transformers, can handle the unconnected features of fingerprints  [31, 32]. Thus, we assessed the efficiency of the back-conversion of fingerprints to molecules to overcome the significant limitations of structural fingerprints that preclude their implementation in NLP models. For this purpose, we employed a translation-based system, namely the transformer architecture, to decode fingerprints accurately into lossless molecular representations. We aim to demonstrate that the reconstruction of molecules from molecular fingerprints is a practical and highly accurate approach for various chemical applications. Finally, we illustrate our approach using thirteen structural fingerprint examples, classified into five main categories. We show that certain fingerprints can be used directly in an NLP setting as alternatives to SMILES and SELFIES representations.

## Results and discussion

### **Structural fingerprint representations**

Structural fingerprints were obtained from RDKit [40] implementations. They can be classified into five main groups, as reported in Table 1 along with the corresponding sequence lengths and vocabulary size information. We generated thirteen different fingerprints for our analysis. Binary variants of the selected fingerprints were hashed to a fixed size of 2048, except for Avalon. Fingerprints were optimized based on their parameters to yield similar sequence lengths when necessary. We omitted sparse versions of atom pairs and ECFP4 from this calculation because the vocabulary space covered, and thus the token size, was considerably large.

1. *Predefined substructure* MACCS keys [41] converts a molecule into a bit vector with a fixed size of 166, in which each bit records the presence of a feature obtained from a predefined dictionary of SMARTS patterns [42].
2. *Paths and feature classes* The Avalon enumerates paths and feature classes. We refer the reader to Gedeck et al. [43] for a thorough explanation of paths and feature classes covered.
3. *Path-based* The RDKit fingerprint is very similar to the Daylight fingerprint [42]. Hashed branched and linear subgraphs of size 4 were used. In both cases, the minPath and maxPath parameters were set to two and four, respectively. The hashed variant of the atom pair fingerprint encodes all pairs of atoms with their environments and their bond distances  [44]. Here, it was used with the following parameters: minLength=1, and maxLength=6.
4. *4-atom-paths* Topological torsion [45] encodes sequences of four bonded atoms, so that the generated set of substructures has a local character. It was used along with its hashed variants.
5. *Circular* ECFPx [35] enumerates circular atom environments, defined as topological neighborhood fragments, up to a selected radius (x). The set of all circular fragments, that is atom environments, is denoted as AEs. Feature-class fingerprints FCFPx include pharmacophoric features as invariants.

### Model overview

In this study, we employed Transformer [46], a model architecture with a multi-head attention mechanism for each unit. Transformer-based models can achieve highly successful translation quality compared to generic seq-2-seq methods [13, 16, 17, 32], thanks to attention units allowing the model to learn global dependencies between inputs and outputs. In addition, the attention mechanism eliminates the dependence on the order of the input sequence. Therefore, the models yield the same sequence of outputs regardless of the spatial connections between the tokens. This property of the attention mechanism renders Transformer-based models suitable for investigating fingerprint-to-molecule conversion.

**Fig. 1:**
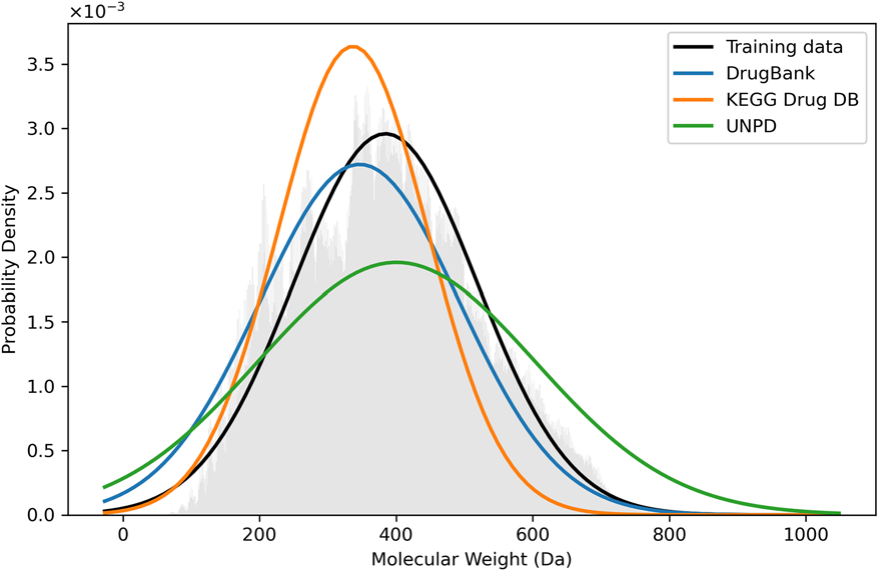
The normalized molecular weight distribution of our training dataset along with several drug and natural product libraries such as KEGG DRUG Database, DRUGBANK and Universal Natural Product Database (UNPD). The training dataset consisted of five million small- and medium-sized molecules of approximately 50 heavy atoms or less that maximally represent available drug-like chemical space

Translation-based algorithms require a large corpus of diverse translation pairs for an effective translation. For this purpose, we selected the ChEMBL [47] (2.08 M) dataset and extended it to include PubChem [48] compounds by maximizing the variety of atom-types based on the atomic environments. Atom-types refer to the features obtained by sparse ECFP of radius zero. This resulted in 5,050,000 small- and medium-sized molecules (those with 50 heavy atoms or less) that maximally represent available drug-like chemical space, considering that most current drugs are small organic molecules of natural or synthetic origin [49]. Figure 1 illustrates the normalized molecular weight distribution of our training dataset, along with several drug and natural product libraries. From this large pool, we randomly selected and separated 50,000 molecules for testing purposes. To obtain more realistic results, we used a challenging dataset, which retains the stereochemical information. However, we note that most of fingerprints in RDKit do not account for stereochemistry.

### Model performance

**Fig. 2:**
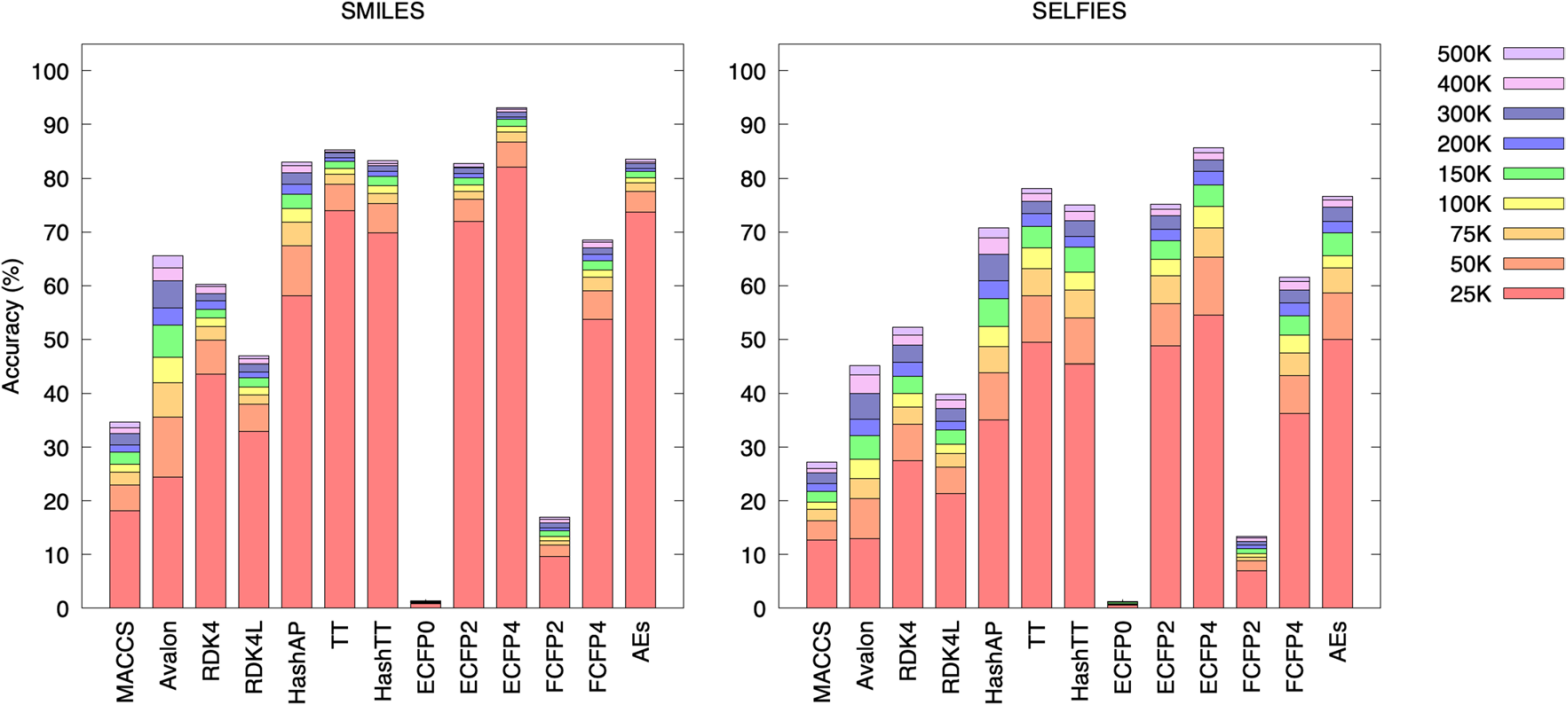
Conversion accuracy of each structural fingerprint to SMILES (left) and SELFIES (right) demonstrated using cumulative column-stacked bar plots along with the number of training steps, from 25K to 500K steps (right color map). The results are based on the Tanimoto exactness, the percentage of $T_\text{c} = 1.0$ reconstructions, computed periodically during training with a sparse form of an extended connectivity fingerprint (ECFP) of radius 1. Each bar represents the progress over the iterations for the given step intervals

The conversion accuracy of each structural fingerprint into unique molecular representations, namely SMILES and SELFIES strings, is illustrated in Fig. 2. The SMILES conversion demonstrated more favorable results in terms of accuracy compared to the SELFIES conversion. In both translation attempts, the top-performing molecular representation was ECFP4. The highest accuracy reached 93.1%, indicating that the model reflects an optimal level of fragment specificity within a fixed-length vector. Alongside the performance of ECFP4, TT, HashAP, and AEs yielded competitive accuracy, whereas the worst performance was observed in MACCS, omitting ECFP0. It should be noted that ECFP0 attempts to represent five million molecules using only 100 tokens so that the produced fragments are overly-general. ECFP0 did not function well in this translation task. Additionally, sparse versions perform better than hashed variants of the same fingerprint, as in the cases of the TT-HashTT and AEs-ECFP2 pairs.

The performances of the structural fingerprints for the SMILES and SELFIES reconstruction showed different dynamics during training. Near-convergence was achieved at a lower number of steps for SMILES compared to SELFIES (learning was quicker, as evident from the relative bar heights after 100 K steps; see Fig. 2). Accordingly, the SMILES grammatical structure can be easily learned, compensating for the fragility of the representation. On the other hand, the decrease in the overall accuracy and the necessity for a more significant step size to reach convergence of SELFIES indicated that the correlations between the fingerprints and SELFIES tokens were weaker than those between the fingerprints and SMILES tokens. The performance of Avalon in the SELFIES prediction differed from the general performance trend, which may be due to its unusual cumulative distribution function (CDF).

**Fig. 3:**
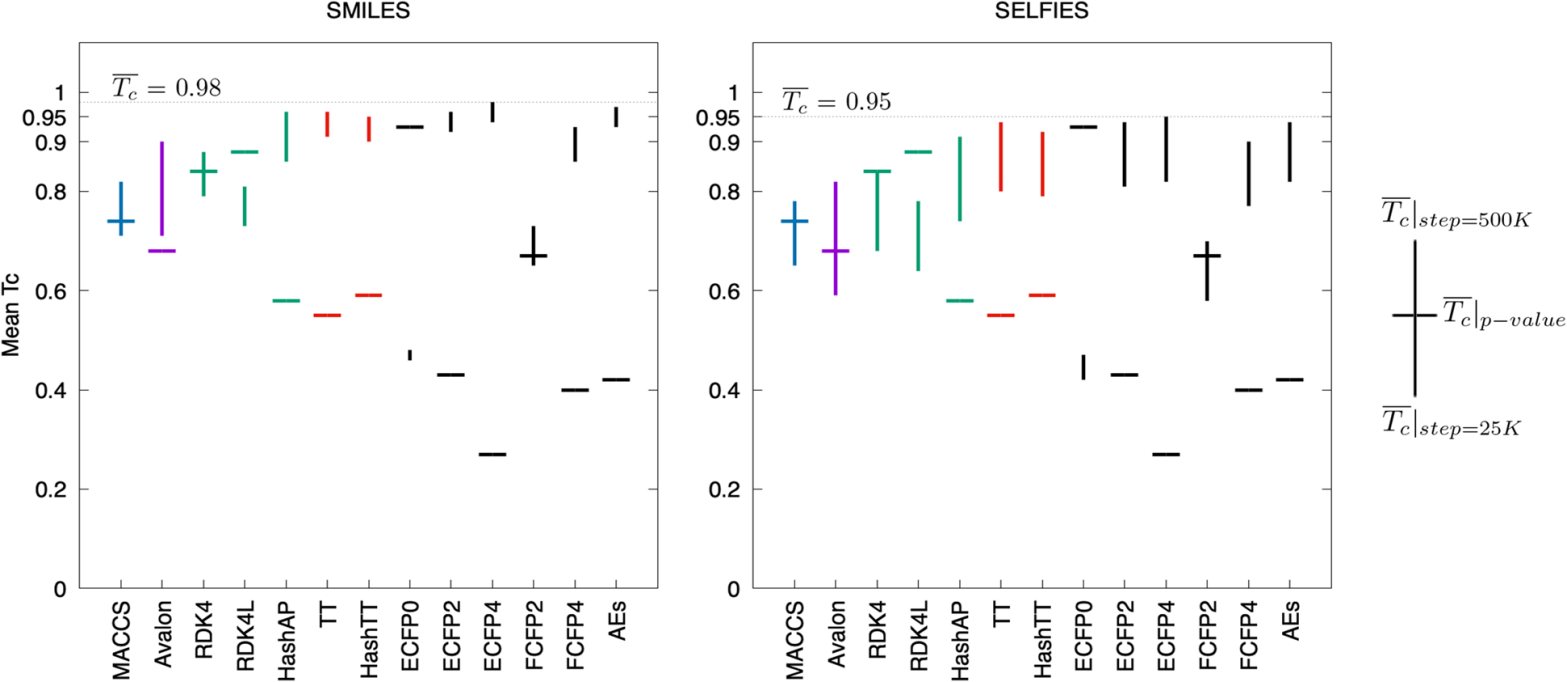
Mean Tanimoto coefficients for each type of conversion along with the reference significance score to assess the actual performance of structural fingerprints. Horizontal lines represent the similarity values of each fingerprint corresponding to a p-value of 0.01. Vertical lines show the continuum, which starts at 25K step and ends with convergence

The mean Tanimoto score ($\hbox {T}_{\textrm{c}}$) is important as it reflects the overall conversion quality. However, similarity metrics generally have different scales for different types of fingerprints. Therefore, it is not ideal to rationalize a specific similarity value as a performance evaluation indicator for various fingerprints. A global comparison of all fingerprints within a fair framework is possible only when the similarity value corresponding to a reference significance score is presented. Considering this, we generated the CDFs of all fingerprints and obtained $\hbox {T}_{\textrm{c}}$ values with a significance of 0.99. Figure  3 illustrates the mean $\hbox {T}_{\textrm{c}}$ scores (vertical lines) within the training step interval [25K-500K] coupled with a fixed p-value of 0.01 (horizontal lines). The small horizontal lines in the Figure 2 were determined for each fingerprint using the method proposed by Vogt and Bajorath [50] to model the distribution of similarity values for various fingerprints in RDKit. These lines represent Tanimoto coefficients for a p-value of 0.01, which allowed us to assess the level of learning.

Lower $\hbox {T}_{\textrm{c}}$ values for the reference significance score, and higher mean $\hbox {T}_{\textrm{c}}$ values at convergence were observed as characteristics of high-performing fingerprints (ECFP4, ECFP2, FCFP4, AEs, HashAP, TT and HashTT). As shown in Fig. 3, the ECFP4-SMILES conversion yielded the best overall result, with a mean $\hbox {T}_{\textrm{c}}$ of 0.98. AEs was the next in terms of performance, having a mean $\hbox {T}_{\textrm{c}}$ of 0.97. The performances of HashAP, TT, and HashTT were comparable to that of AEs, with mean $\hbox {T}_{\textrm{c}}$ scores of 0.96, 0.96, and 0.95, respectively. In contrast, the RDKit variants-SELFIES conversion performed poorly relative to the other path-based fingerprints.

**Fig. 4:**
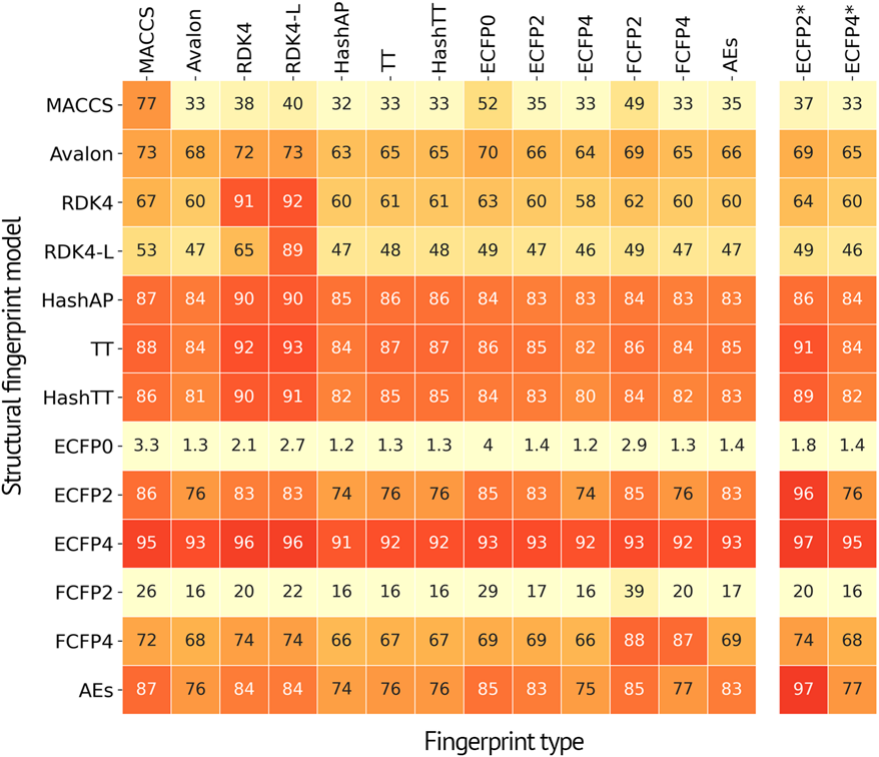
Percentages of reconstructed SMILES strings from a source fingerprint (y-axis) with $T_\text{c}=1.0$, the Tanimoto exactness, computed with the respective fingerprints (x-axis). The consistent values across a row reflect the robustness and high quality of reconstructed SMILES strings, while significant variations of values represent the fingerprint bias in $T_\text{c}$ calculation ECFP2$^{*}$ and ECFP4$^{*}$ represent explicit bit versions

Predictive performance is often susceptible to bias if the fingerprints representing the input sequences are used to compute the similarity score. To minimize the selection bias, multiple fingerprints were used, as listed in Table 1. The Tanimoto exactness of each model, the percentage of predictions under the condition that $\hbox {T}_{\textrm{c}}$ equals unity, was computed across 15 different fingerprints (by including explicit bit vector type of the ECFP2 and ECFP4), and is presented as a matrix in Fig. 4. This approach was essential to our assessment as it decoupled the robustness of the models from the effectiveness and bias of the fingerprints. The enhanced prediction accuracies of MACCS, RDK4, RDK4-L, and ECFP2 fingerprints confirmed the fingerprint dependency of the results. Figure 4 highlights the high performance and robustness of the ECFP4-SMILES model. The true performance of each model averaged over 15 fingerprints is presented in Table 2. Ultimately, our top-performing models, such as ECFP4, TT and its hashed variant, HashAP, ECFP2 and AEs, performed similarly regardless of the choice of similarity metric. An analysis of the fingerprint dependency of SELFIES is shown in the Additional File 1: Figure S2.

### Breakdown of the top-1 accuracy

A complete breakdown of the top-1 accuracy results over the 50 K test set for the top-performing structural fingerprints is presented in Table 3, wherein the total accuracy is given based on Tanimoto exactness. We further separated the total accuracy into major components, using a simple string comparison. Here, we note that identical structures based on the Tanimoto metric can be categorized depending on whether they are sourced from identical strings, stereochemistry, canonicalization, or other characteristics, including chain length and symmetry properties. The invalidity rates and mean Tanimoto scores are listed in Table 3.

A large fraction of our test set (i.e., $\sim$30%) incorporates stereochemistry, and the obtained results indicate that the models account for stereochemical information. However, they struggle to achieve an accurate picture of relative atom orientations. Indeed, for the best-performing fingerprint, ECFP4, the stereochemical errors equaled $\sim$20%. Therefore, we examined the stereochemically-inconsistent predictions by removing the stereochemical information to determine whether these predictions were string-exact relative to the ground truths. In most cases, the models treat reverse (or opposite) stereochemistry as cis/trans or clockwise/anti-clockwise. Moreover, predictions featuring stereochemistry also existed even when the ground truths possessed no stereocenters, or vice versa.

Our dataset was not subjected to canonicalization before training to investigate the full capacity of the SMILES representation. Our models could produce noncanonical instances of ground-truth SMILES representations, and the rates of predicting chemically equivalent SMILES representations varied from 1.6 to 4.8%, depending on the fingerprint type. In addition, it should be noted that the Kekule forms play an important role in non-canonical predictions because switches in the Kekule representations can alter SMILES enumerations. SELFIES provided robust conversions regarding invalidity rates, with no invalid cases, as expected. Furthermore, SMILES performed comparably well, with only  0.2-$-$0.3% invalidity rates. Representative predictions displaying the changes in stereochemistry, kekule forms, and enumerations are provided in Additional File 1: Table S1.

### Interpretability

**Fig. 5:**
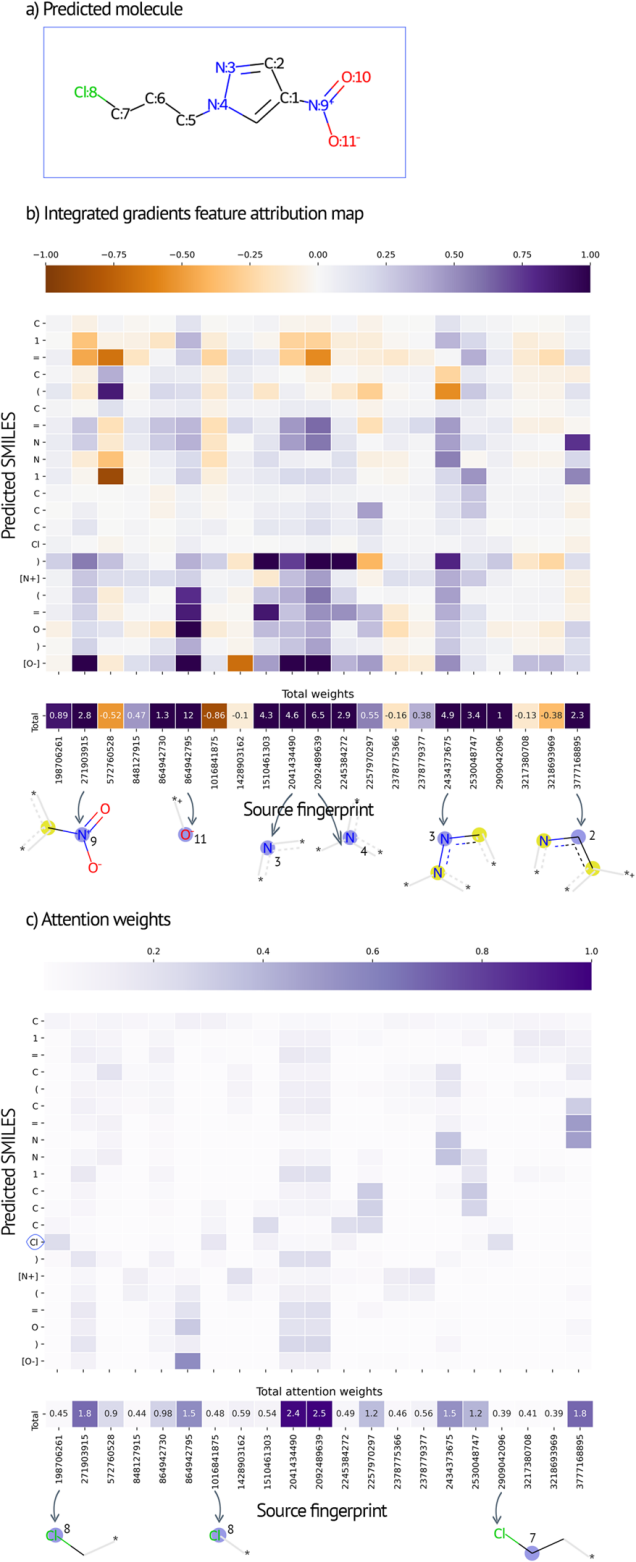
Correlated features of the **a** predicted SMILES given with atomic indices obtained by **b** integrated gradients and **c** attention weight matrices. The most salient fragments (atom indices attached to the central atoms for easy recognition) are interpreted column-wise and row-wise

Translation-based models require a detailed quantitative study of the relationships between the translated pairs. To establish a thorough explanation of the model, we evaluated the correlated features obtained using the integrated gradients and attention weights, commonly used to explain the relationship between tokens (Fig. 5). As a form of gradient-based feature importance measure, integrated gradients reveal relevant features more reliably than attention weights. Recent findings showed that attention weights are often uncorrelated with gradient-based methods [51, 52]. Therefore, we recognized attention weights as a valuable supplementary tool to address the interpretability problem. Although the interpretation of attribution matrices for each combination is highly intricate, an explainable path exists between the AEs and the reconstruction of the SMILES string.

The matrices shown in Fig. 5 can be interpreted in two ways: First, the column-wise approach reflects the effect of an input feature on the prediction. Based on this approach, our results indicated that the high-attribution AEs at positions 9 and 11 were the most salient fragments for predicting the SMILES substring of the nitro groups (Fig. 5b). In particular, the AE at position 11, with a radius of 0, made a decisive contribution specifically to the oxygen atoms of the nitro group because the negatively charged oxygen is in resonance with the geminal oxygen. Second, the row-wise approach reflects salient input features attributed to a specific part of the prediction. For example, the higher attention values in the row of chlorine atoms (Fig. 5c) highlight three atomic environments, all containing chlorine, including the central atoms at radii 0 and 1.

## Conclusion

Structural fingerprints were exploited as alternatives to unique molecular representations. We successfully rebuilt the molecules with a high level of precision, that is, >90% for the top-performing fingerprints. Consequently, structural fingerprints can be used as strong representational tools in chemistry-related NLP applications after restoring the connectivity information lost during fingerprint transformation. Therefore, our diverse selection of fingerprints provided an unbiased examination of the overall conversion performance. Our results indicated that AEs, ECFP4, topological torsion, and atom-pair fingerprints are ideal candidates for developing NLP tools with molecules.

In this study, a complete breakdown of the accuracy per fingerprint class is presented in detail. Such an analysis provides invaluable insights into the critical factors affecting the conversion process, such as stereochemistry, which was a noticeable limitation of the model proposed herein. As this model has struggled to treat stereochemistry, additional research is required to fully address this issue. Moreover, we assessed the interpretability of our conversion approach by evaluating the methods that compute and extract the most salient features for prediction. The attribution maps revealed that the model focused on the correct fragments for reconstructing the molecule. Finally, our findings could help improve the quality of outcomes by offering ways to develop more efficient chemical models in the fields of deep generative modelling and neural machine translation.

## Method

### Training

The Pytorch [53] Distributed Data-Parallel Training (DDP) module was employed to train our models. Each model was trained with two GPUs up to a 500 K step, which denotes the number of times the optimizer updates the parameters of the model. The hyperparameters of the models were set similar to ones used in the original Transformer publication [46]. The encoder and decoder used a stack of six identical layers consisting of eight heads with a 512 dimensional multi-head attention mechanism, followed by a 2048 dimensional fully connected feed-forward layer. In contrast, we used a normalization layer before each sub-layer, and with the outputs of the encoder and decoder by following The Annotated Transformer [54]. A dropout layer, with a dropout rate of 0.1, was applied to the output of each sub-layer to avoid overfitting.

Even though the attention mechanism is a permutation-invariant operation and fingerprint features are unconnected, we did not remove the positional encoding in our final models because a previous study [39] stated that it was preferable over non-positional encoding. We had tested this claim by training our models without positional encoding. The results of our models with positional encoding showed slightly better performance (in a range of 0.2$-$0.9 percent) compared to those without positional encoding, consistent with the previous findings [39, 55] Though not significant, what considered at positional encoding was the default order of fingerprints features, e.g., index-order for hashed fingerprints. In addition, a zero-redundancy optimizer (ZeRO) [56] with the Adam algorithm was employed to optimize the parameters of the models. This was done to improve the training speed by eliminating memory redundancies during data- and model-parallel training. A negative log-likelihood function was used as the loss function.

We set the number of tokens in one batch to 8000 per GPU. Owing to hardware limitations, this number could not be exceeded. For a fair comparison of the fingerprints, the batch size was specified based on the average number of tokens in one batch, provided that the number of sentence pairs in one batch varied according to the fingerprint sequence length. To extend the performance of the standard transformer implementation [46], we experimented with several learning-rate schedulers. In addition to testing stochastic gradient descent with warm restarts [57], we designed a decayed variant of the cyclic learning rate because the importance of scheduling is well emphasized by Karpov [58]. The behaviors of the schedulers are shown in Additional File 1: Figure S1. The cyclic learning scheduler was ultimately selected as the most appropriate scheduler because it provided a slightly superior performance compared to the other techniques. For the cyclic rate scheduler, the constant factor parameter and the warm-up step size were set to 5 and 5000, respectively. The learning rate decreased from 0.001 to 3.9e$-$12 at each 25 K step and increased to its maximum again.

### Evaluation

Conversion efficiency was evaluated using Tanimoto similarity matching. Further breakdown of the results was achieved by introducing simple string matching. The widely used Tanimoto coefficient, which operated on the sparse Morgan fingerprint, was selected as the similarity metric to represent the main results. Pairwise similarities between the predictions and ground truths were computed at the end of each 25K step for each pair present in the test set. Top-1 predictions were used to report the conversion accuracy, and the Python package ccbmlib [50] was employed to facilitate the generation of similarity value distributions for all fingerprints.

**Table 1:** Translation-related statistics regarding the domain-specific datasets generated by the structural fingerprints used for the performance analysis, together with the targeted molecular representations, SMILES, and SELFIES

Abbreviations	Description	Dim	Sequence length		Token size
			Ave.	Max	
**Predefined substructures**					
MACCS		166	50	107	160
**Paths and feature classes**					
Avalon	Hashed	512	182	470	516
**Path-based**					
HashAP	Atom pair - hashed	2048	92	273	1998
RDK4	RDkit fingerprint - hashed	2048	83	288	2052
RDK4-L	RDK4 - with no branch	2048	58	209	2052
**4-atom-paths**					
TT	Topological torsion	sparse	32	124	54973
HashTT	TT - hashed	2048	31	118	2052
**Circular**					
AEs	Morgan radius 1	sparse	29	65	54076
ECFP0	Morgan radius 0 - hashed	2048	10	25	100
ECFP2	Morgan radius 1 - hashed	2048	28	64	2052
ECFP4	Morgan radius 2 - hashed	2048	47	103	2052
FCFP2	Feature-class of ECFP2	2048	20	51	1576
FCFP4	Feature-class of ECFP4	2048	36	86	2052
**Unique Representation**					
SMILES	Tokenized atom-wise		51	125	109
SELFIES	Generic tokenization		44	127	205

**Table 2:** Overall performance (%) of fingerprint decoders, computed as the average Tanimoto exactness score across 15 fingerprintsOverall performance (%) of fingerprint decoders, computed as the average Tanimoto exactness score across 15 fingerprints

	MACCS	Avalon	RDK4	RDK4L	HashAP	TT	HashTT	ECFP0	ECFP2	ECFP4	FCFP2	FCFP4	AEs
SMILES	39.6	67.3	65.2	51.6	85.1	86.6	84.6	1.9	80.8	93.6	20.3	71.7	81.3
SELFIES	31.2	46.6	56.7	44.1	72.6	79.5	76.4	1.6	73.6	86.2	16.3	64.7	75.0

**Table 3:** Detailed breakdown (%) of top-1 accuracy on 50 K test set for the top-performing structural fingerprints belonging to five sub-categories

Representation	Components	MACCS	Avalon	HashAP	TT	AEs	ECFP4
SMILES	$T_c = 1.0$	34.7	65.6	83.1	85.2	83.5	93.1
	String exact	22.3	44.7	58.7	57.8	52.1	64.6
	Stereo	8.2	14.9	19.2	19.2	18.0	21.2
	Non-canonical	1.6	3.5	4.3	4.2	3.7	4.8
	Others	2.6	2.6	0.8	4.0	9.6	2.5
	Invalid	0.2	0.4	0.3	0.3	0.3	0.2
	$\overline{T_{c}}$	81.9	90.5	95.5	96.3	96.7	98.1
SELFIES	$T_c = 1.0$	27.2	45.2	70.7	78.0	76.6	85.6
	String exact	17.7	31.3	50.9	54.0	49.1	60.5
	Stereo	5.9	9.3	15.2	16.7	19.9	18.5
	Non-canonical	1.5	2.8	4.0	4.1	3.6	4.7
	Others	2.2	1.7	0.6	3.3	8.0	1.9
	Invalid	No invalid predictions					
	$\overline{T_{c}}$	77.8	81.5	90.7	93.9	94.4	95.1

## Supplementary Information

**Additional file 1: Figure S1.** We tried four different learning rate schedulers. CylicLR in reference to Karpov et al., its decay variant that is designed in this study, the scheduler used in standard Transformer paper, and stochastic gradient descentwith warm restarts (SGDR). The cyclic learning scheduler was selected due to its slightly superior performance compared to the other techniques. The constant factor parameter and the warm-up step size were set to 5 and 5000, respectively. The learning rate decreased from 0.001 to 3.9e-12 at each 25K steps and jumped to its maximum again.** Figure S2.** Each cell shows the Tanimoto exactness (%) of selected fingerprint transformation to SELFIES (y-axis) computed at the respective fingerprint encodings. The consistency in color code reflects the robustness, while the jumps represent the effect of selection bias. ECFP2* and ECFP4* represent explicit bit versions.** Table S1.** Case 1: Ground Truth has stereo information but prediction has in reverse form. Case 2 : Ground Truth has stereo information but prediction does not. Case 3 : Ground Truth has no stereo information but prediction does. Case 4 : Enumerations are different. Case 5 : Ground Truth is not in kekulized form but prediction is.

## Data Availability

The data that support the findings of this study, the source code, and the associated trained models are all available at the MolForge GitHub repo: https://github.com/knu-lcbc/MolForge.
