# Correction: Reconstruction of lossless molecular representations from fingerprints

**DOI:** 10.1186/s13321-023-00739-3

**Published:** 2023-07-26

**Authors:** Umit V. Ucak, Islambek Ashyrmamatov, Juyong Lee

**Affiliations:** 1 Research Institute of Pharmaceutical Science, College of Pharmacy, Seoul National University, 1 Gwanak-ro, Gwanak-gu, Seoul, 08826 Republic of Korea; 2 Department of Chemistry, Kangwon National University, Chuncheon, 24341 Republic of Korea; 3 Molecular Medicine and Biopharmaceutical Sciences, Graduate School of Convergence Science and Technology, Seoul National University, 1 Gwanak-ro, Gwanak-gu, Seoul, 08826 Republic of Korea

**Correction** **:** **Journal of Cheminformatics (2023) 15:26** **https://doi.org/10.1186/s13321-023-00693-0**

Following publication of the original article [1], the authors requested to correct the funding number NRF-2019M3E5D4066898 to NRF-2022M3E5F3081268.

**Funding**

This work was supported by National Research Foundation of Korea (NRF) Grants funded by the Korean government (MSIT) (Nos. NRF-2022M3E5F3081268, NRF-2022R1C1C1005080 and NRF-2020M3A9G7103933 to I.A. and J.L.). This work was also supported by the Korea Environment Industry & Technology Institute (KEITI) through the Technology Development Project for Safety Management of Household Chemical Products, funded by the Korea Ministry of Environment (MOE) (KEITI:2020002960002 and NTIS:1485017120 to U.V.U. and J.L.).

The original article has been corrected.

## References

[1] Ucak UV, Ashyrmamatov I, Lee J (2023). Reconstruction of lossless molecular representations from fingerprints. J Cheminform.

