# Supplementary material for: Reconstruction of lossless molecular representations from fingerprints
Source: J Cheminform. 2023 Feb 23;15:26. doi: 10.1186/s13321-023-00693-0 (PMC9948316; doi:10.1186/s13321-023-00693-0)
Supplement: Supplementary file 1 — Additional file 1: Figure S1. We tried four different learning rate schedulers. CylicLR in reference to Karpov et al., its decay variant that is designed in this study, the scheduler used in standard Transformer paper, and stochastic gradient descentwith warm restarts (SGDR). The cyclic learning scheduler was selected due to its slightly superior performance compared to the other techniques. The constant factor parameter and the warm-up step size were set to 5 and 5000, respectively. The learning rate decreased from 0.001 to 3.9e-12 at each 25K steps and jumped to its maximum again. Figure S2. Each cell shows the Tanimoto exactness (%) of selected fingerprint transformation to SELFIES (y-axis) computed at the respective fingerprint encodings. The consistency in color code reflects the robustness, while the jumps represent the effect of selection bias. ECFP2* and ECFP4* represent explicit bit versions. Table S1. Case 1: Ground Truth has stereo information but prediction has in reverse form. Case 2 : Ground Truth has stereo information but prediction does not. Case 3 : Ground Truth has no stereo information but prediction does. Case 4 : Enumerations are different. Case 5 : Ground Truth is not in kekulized form but prediction is. [file 13321_2023_693_MOESM1_ESM.pdf]

Additional Files

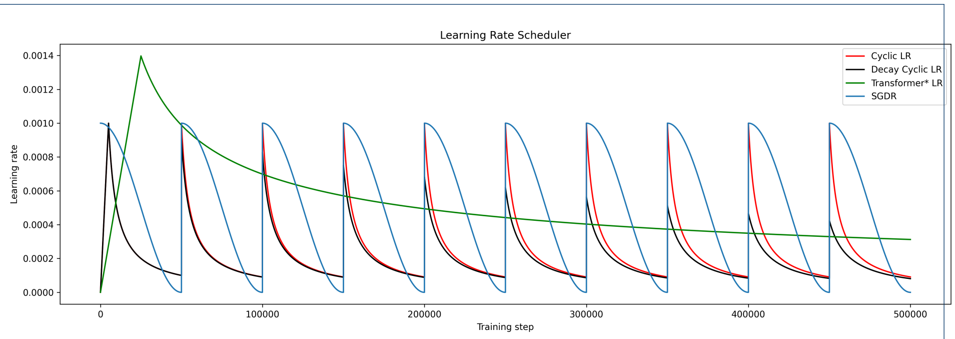

Supplementary Figure 1: We tried four different learning rate schedulers. Cylic LR in reference to Karpov et al., its decay variant that is designed in this study, the scheduler used in standard Transformer paper, and stochastic gradient descent with warm restarts (SGDR). The cyclic learning scheduler was selected due to its slightly superior performance compated to the other techniques. The constant factor parameter and the warm-up step size were set to 5 and 5000, respectively. The learning rate decreased from 0.001 to 3.9e-12 at each 25K steps and jumped to its maximum again.

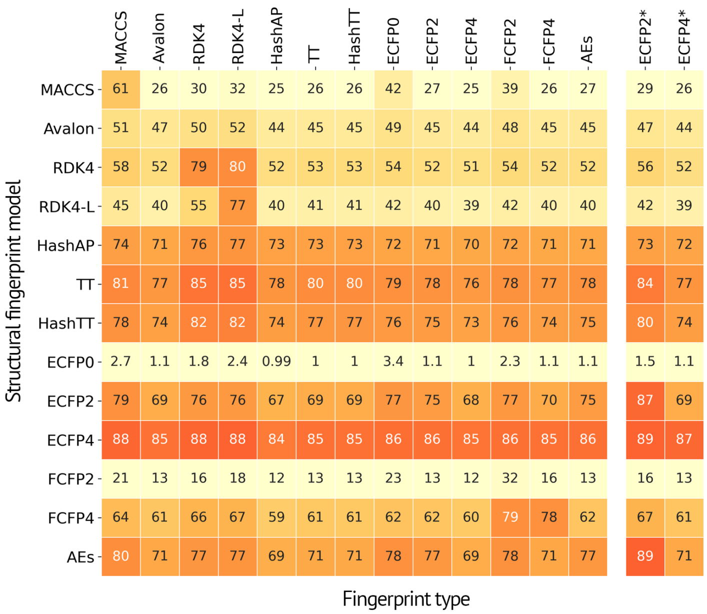

Supplementary Figure 2: Each cell shows the Tanimoto exactness (%) of selected fingerprint transformation to SELFIES (y-axis) computed at the respective fingerprint encodings. The consistency in color code reflects the robustness, while the jumps represent the effect of selection bias. ECFP2* and ECFP4* represent explicit bit versions.

Supplementary Table 1: Case 1 : Ground Truth has stereo information but prediction has in reverse form. Case 2 : Ground Truth has stereo information but prediction does not. Case 3 : Ground Truth has no stereo information but prediction does. Case 4 : Enumerations are different. Case 5 : Ground Truth is not in kekulized form but prediction is.

	Truth	Prediction
1	Truth	C1=CC(=CC=C1/C(=C\\C#N)/Cl)Cl
	Prediction	C1=CC(=CC=C1/C(=C/C#N)/Cl)Cl
2	Truth	CCCCN1C(=O)NN=C1S[C@@H](C)C(=O)C2=C(C(=C(N2)C)C(=O)OC)C
	Prediction	CCCCN1C(=O)NN=C1SC(C)C(=O)C2=C(C(=C(N2)C)C(=O)OC)C
3	Truth	CC(C(=O)NC1=CC=CC=C1C(=O)C2=CC=CC=C2)NC3=CC4=C(C=C3)OCO4
	Prediction	C[C@@H](C(=O)NC1=CC=CC=C1C(=O)C2=CC=CC=C2)NC3=CC4=C(C=C3)OCO4
4	Truth	N1C(C=C(N=C1NC#N)C)(C)C
	Prediction	CC1=CC(NC(=N1)NC#N)(C)C
5	Truth	c1(ccc2c(c1)c(cc(n2)c1ccccc1)N/N=C/c1ccc(cc1)N(C)C)OC
	Prediction	CN(C)C1=CC=C(C=C1)/C=N/NC2=CC(=NC3=C2C=C(C=C3)OC)C4=CC=CC=C4
